# Challenges in the Diagnosis of Leak After Sleeve Gastrectomy: Clinical Presentation, Laboratory, and Radiological Findings

**DOI:** 10.1007/s11695-020-05008-y

**Published:** 2020-10-06

**Authors:** Mohammad Al Zoubi, Nesreen Khidir, Moataz Bashah

**Affiliations:** 1 General Surgery Department, Hamad Medical Corporation, Hamad General Hospital, P.O. Box: 3050, Doha, Qatar; 2 Bariatric and Metabolic Surgery Department, Hamad Medical Corporation, Doha, Qatar; 3 Weil Cornel Medical College, Doha, Qatar

**Keywords:** Diagnosis of leak post sleeve gastrectomy, Post LSG leak, Misdiagnosis of leak post LSG, Post LSG complications

## Abstract

**Background:**

The presentation of leak after laparoscopic sleeve gastrectomy (LSG) is variable. A missed or delayed diagnosis can lead to severe consequences. This study presents our experience: the clinical presentations, laboratory, and radiological findings in patients with leak after LSG.

**Methods:**

A retrospective review of patients who were diagnosed and treated as leak after LSG at our center (January 2012–November 2019).

**Results:**

Eighty patients developed leak: 68 (85%) after primary LSG, 6 (7.5%) after Re-LSG and 6 (7.5%) after band removal to revisional LSG. Mean age 35.9 ± 10 years. The diagnosis was within 18 ± 14 days after surgery. Five (6.3%) patients were diagnosed during the same admission. Only 29.3% of patients were diagnosed correctly from the first visit to the ER. Most were misdiagnosed as gastritis (49%) and pneumonia (22.6%). Thirty-four patients (45.3%) were diagnosed correctly at the third visit. The most common presenting symptoms were abdominal pain (90%), tachycardia (71.3%), and fever (61.3%). The mean white blood cells (WBCs) count was 14700 ± 5900 (cells/mm^3^), c-reactive protein (CRP) 270 ± 133 mg/L, lactic acid 1.6 ± 0.85 mmol/L, and albumin 30.3 ± 6.6 g/L. The abdominal CT scans revealed intraabdominal collection in 93.7% of patients, extravasation of contrast in 75%, and pleural effusion in 52.5%. Upper gastrointestinal contrast study (UGIC) showed extravasation of contrast in 77.5% of patients.

**Conclusion:**

Abdominal pain, tachycardia, or fever after LSG should raise the suspicion of a leak. CT scan of the abdomen and UGIC study detected leaks in 75% and 77.5% consecutively. Only 29.3% of patients were diagnosed correctly as a leak from the first visit to the ER.

## Introduction

Despite the decreasing worldwide incidence over time, gastrointestinal leak remains a significant cause of morbidity and mortality after bariatric surgeries [1]. Leakage rates after laparoscopic sleeve gastrectomy (LSG) were reported ranging from 0.4 to 2.7% in primary procedures and 10% in revisional procedures [2–4].

Post LSG leaks can be misdiagnosed, resulting in delayed management and catastrophic consequences [5]. The clinical presentation, signs, and symptoms are highly variable, ranging from asymptomatic to septic shock [6]. Debate exists on what is the best diagnostic modality in diagnosing LSG leak. Though all agreed that early detection is associated with a better outcome, and a high index of suspicion is the cornerstone in the diagnosis [5, 7, 8]. This study shares our experience in treating a large number of LSG leaks at a single center, detailing the clinical presentations, laboratory, and radiological findings.

## Objectives

The objective of this study is to determine the clinical presentations, laboratory results, and radiological findings of patients diagnosed with leak post LSG at our center, (January 2012–November 2019).

## Methods

A retrospective data review of patients who were diagnosed and treated as post LSG leak at Hamad Medical Corporation—Qatar (January 2012–November 2019). Our center is a tertiary hospital with a bariatric and metabolic surgery department performing 900–1000 laparoscopic and endoscopic bariatric procedures per year, including around 700 LSG. The hospital is the only referral center in the country for bariatric surgery complications, receiving patients operated in the private sector and abroad. Routinely, when patients arrive at the emergency room (ER), they are first worked up and managed by ER physicians. Once a surgery-related complication is identified, only then the bariatric surgery team will be involved. The data of wrong diagnoses and repeated visits to the ER were retrieved from the patients’ electronic medical records. All leak patients were included in this study, regardless of where they had been operated.

The leak was diagnosed by radiological findings of oral contrast extravasation either in CT scan and upper gastrointestinal contrast (UGIC) fluoroscopy study or by endoscopic findings of fistulous opening. Our protocol in diagnosing and managing LSG leaks was published earlier [2].

Patients’ demographics including (age, BMI, gender, comorbidities, time of the leak, type of surgery), clinical symptoms and signs, laboratory findings, radiological imaging, number of visits to ER, and misdiagnoses were reviewed. The study was approved by the medical research center “IRB approval number 16208/16”.

### Surgical Technique

Laparoscopic CO2 pneumoperitoneum was created by optic technique through a supraumbilical incision. Three more dilated trocars were introduced. Nathanson liver retractor was routinely utilized. The division of gastrosplenic ligament was performed along the greater curvature 4 cm from the pylorus up to the left diaphragmatic crus with ultrasonic shears. The stomach was then mobilized and divided along the lesser curvature from antrum (4 cm from pylorus) up to the angle of His using a buttressed (SeamGuard) linear 60-mm stapler over the calibration tube (Mid-sleeve 38Fr) introduced into the stomach. The specimen was removed through the umbilical port. The procedure was concluded with a methylene blue leak test in all patients.

### Statistical Analysis

Descriptive statistics were provided as frequencies and proportions in case of categorical variables or means and standard deviations in case of continuous variables. Statistical significance was set at *P* < 0.05. No sample size calculation was done; the study is a retrospective review. Statistical analysis was performed using STATA 15 software. Data were available in > 85% of patients. No follow-up rate was calculated since all the reviewed data was during the same admission.

## Results

Eighty patients developed leak: 68 (85%) after primary LSG, 6 (7.5%) after Re-LSG, and 6 (7.5%) after band removal to revisional LSG. Out of the eighty patients, only twenty patients (25%) had LSG done at our center. The patients’ mean age was 35.9 ± 10 years, and mean BMI was 42 ± 9.5 kg/m^2^. Patients were thirty males (37.5%) and fifty females (62.5%). The demographic data were represented in Table 1.

**Table 1:** Demographic data of patients with post laparoscopic sleeve gastrectomy leak

Variable	Value
Age (years, M ± SD)	35.9 ± 10.3
BMI (kg/m^2^, M ± SD)	42 ± 9.5
Female (*n*, %)	50 (62.5%)
Postop day of presentation (M ± SD)	18 ± 14
DM (*n*, %)	16 (20%)
HTN (*n*, %)	19 (23.7%)
DM and HTN (n, %)	10 (12.5%)
Hypothyroidism (*n*, %)	5 (6.3%)
Timing of diagnosis (*n*, %)	
Early (0–2 days)	1 (1.25%)
Intermediate (3–14 days)	39 (48.75%)
Late (> 14 days)	40 (50%)

*M* Mean; *SD* standard deviation; *BMI* body mass index; *n* number; *ER visits* number of prediagnosis visits to the emergency room; *DM* diabetes mellitus; *HTN* hypertension, *LSG*: laparoscopic sleeve gastrectomy

The diagnosis was within 18 ± 14 days after surgery. No patients had positive intraoperative methylene blue leak test, and all patients were diagnosed postoperatively. Five patients (6.3%) were diagnosed at the same admission while 75 patients (93.8%) were diagnosed after discharge. Only 29.3% of patients were diagnosed correctly as a leak from the first visit to the ER and 25.3% after two visits. Most patients were misdiagnosed as gastritis 49%, followed by pneumonia in 22.6%. Table 2 shows the list of misdiagnoses at ER visits. The most common presenting signs and symptoms were abdominal pain (*n* = 72, 90%), tachycardia (*n* = 57, 71.3%), and fever (*n* = 49, 61.3%). Table 3 depicts the details of the clinical presentation and laboratory findings. Most patients had abnormal white blood cells (WBCs) and C-reactive protein (CRP) levels. The CRP had a 95% confidence interval (CI) between 228.4–312.4 mg/L. Table 4 shows the imaging findings of CT scan and upper gastrointestinal contrast meal study.

**Table 2:** Details of incorrect diagnosis for patients with post sleeve gastrectomy leaks who had more than one ER visits to the emergency (53 patients)

Incorrect diagnosis	*n* (%)
Gastritis	26 (49.0%)
Pneumonia	12 (22.6%)
Shoulder pain	2 (3.7%)
Pulmonary embolism	2 (3.7%)
Minimal intraabdominal collection	11 (20.7%)

*n* Number of patients

**Table 3:** Details of clinical signs and symptoms and laboratory findings

Variable	Value
Signs and symptoms	
Abdominal pain (*n*, %)	72 (90.0%)
Shoulder pain (*n*, %)	28 (35.0%)
Back pain (*n*, %)	20 (25.0%)
Tachycardia (*n*, %)	57 (71.3%)
Fever (*n*, %)	49 (61.3%)
Shortness of breath (*n*, %)	34 (42.5%)
Nausea (*n*, %)	33 (41.3%)
Vomiting (*n*, %)	28 (35.0%)
Laboratory findings	(M ± SD)
WBCs (cells/mm^3^)	14700 ± 5900
CRP (mg/L)	270 ± 133
Lactic acid (mmol/L)	1.6 ± 0.85
Platelets (10^3/L)	367 ± 128
Albumin (g/L)	30.3 ± 6.6

*n* Number of patients

**Table 4:** Imaging findings of CT scan and upper gastrointestinal contrast meal study

Finding	*n* (%)
CT—Intraabdominal collection	75 (93.7%)
CT—Contrast extravasation	60 (75.0%)
CT—Pleural effusion	42 (52.5%)
CT—Free air	37 (46.2%)
Contrast swallow—Free contrast passage	68 (85.0%)
Contrast swallow—Contrast extravasation	62 (77.5%)

*n* Number of patients; results are based *n* total of 80 patients

The abdominal CT scan revealed collection in 75 patients (93.7%), extravasation of contrast in 60 patients (75%), and pleural effusion in 42 patients (52.5%). The numbers of patients with fat stranding or inflammatory postoperative changes were not reviewed. All patients with extravasation of contrast had an intraabdominal collection as well. Water-soluble contrast swallow showed extravasation of contrast in 62 patients (77.5%). The number of patients who showed gastric fistula opening at endoscopy was out of the scope of this paper.

## Discussion

The diagnosis of gastrointestinal leak after bariatric surgery can be challenging. The patient’s presentation varies according to the type and timing of the leak and the patient’s systemic inflammatory response. Patients with morbid obesity may show uncertain presentations, leading to late diagnosis and potentially catastrophic consequences [5].

Earlier, we described the management of leak as a multimodal process and emphasized the importance of clinical judgment. Most patients developed symptoms after discharge from the hospital. They presented to ER and were assessed by an ER physician. Patients with leak or intraabdominal collection after gastrointestinal surgery usually have abdominal pain, tachypnea, tachycardia, fever, and leukocytosis. However, leak patients after bariatric surgery may present with an unclear picture. Unfortunately, in our patients, only 29.3% (*n* = 22) were diagnosed correctly from their first visit to the ER. Most patients (as shown in Table 2) were misdiagnosed as gastritis (49%) and pneumonia (22.6%). Eleven patients (20.7%) had CT findings of minimal intraabdominal collection or postoperative changes and were sent home. In nineteen patients (25.3 %), a leak was diagnosed correctly at the second visit to ER, and thirty-four patients (45.3%) were diagnosed correctly as a leak at their third visit. Having a large number of misdiagnoses raises the alarm of the importance of clinical judgment by the treating physician “emergency physician in this case”. The role of the ER physicians is pivotal in early detection of the leakage, and it is essential to educate them. Herein, we stress the importance of having a low threshold of suspicion of a leak in patients who do not do well after bariatric surgery.

Authors described tachycardia, fever, and abdominal pain (often radiating to the left shoulder or scapular region) as the most common, but not exclusive, signs of post LSG leak [9–11]. The most common presenting symptoms in our patients were pain, 90% abdominal, 35% shoulder, and 25% back. Some patients suffered from being treated incorrectly for weeks. They had been referred to orthopedics, chest physicians, etc. Unfortunately, the data of timing between the first presentation to the ER and the correct diagnosis were not available. Tachycardia was present in 71.3%, and fever was present in 61.3% of patients. Some agreed that tachycardia was the earliest and constant clinical finding indicating the presence of a gastric leak [9, 12]. The three parameters, abdominal pain (90%), tachycardia (71.3%), and fever (61.3%), were present together in 40 patients (50%). In our opinion, patients with this triad should be managed as leak until proven otherwise. Shortness of breath or tachypnea was significantly present in 42.5% of patients. Nausea and vomiting were present in 33 (41.3%) and 28 (35%) patients, consecutively. Although nausea and vomiting had been reported as ones of the presenting symptoms in leak patients after LSG [13], they are also common in up to 79% of patients in the early postoperative period [14, 15].

In terms of laboratory studies, some authors found that blood investigations including complete blood count (CBC) and (CRP) were neither sensitive nor specific and rarely contributed to making a diagnosis of a leak [5]. At the same time, others found that most leak patients had increased WBC and CRP levels [9]. Inline, our patients’ mean WBCs count was 14700 ± 5900 (cells/mm^3^). Sixty-six patients (82.5%) had abnormally elevated WBCs count. Mean CRP level of 270 ± 133 mg/L. About 95% of patients had their CRP levels between 228.4 and 312.4 mg/L. This second colleagues’ opinion stated “elevated inflammatory markers after LSG and correlation with the clinical picture increase the suspicion for a leak” [9]. Husain et al. [16] found that hypoalbuminemia was a significant risk factor predicting the overall complications after bariatric procedures, including leak. Sixty-one patients (76.2%) had an albumin level below the normal range (35–55) g/L.

CT scan was established as the best noninvasive diagnostic tool for detection and confirmation of a gastric leak [10, 17, 18]. In a multicenter study, authors have noted that CT had the highest detection rate of gastric leaks in up to 86% of patients with (83–93%) sensitivity and (75–100%) specificity [19, 20]. Besides, inclusion of the chest may help to rule out other causes of shortness of breath or tachypnea, such as pneumonia, pulmonary embolism, or pleural effusion [1]. Some leak patients were diagnosed as a leak by CT chest to rule out pulmonary embolism, and accidentally, the lower chest and upper abdomen images revealed findings suggestive of a leak. In our study, contrast extravasation in CT was detected in 75% of patients, while in the UGIC study extravasation was detected in 77.5%. UGIC study was performed routinely in all of our patients with a suspicion of the leak [2], regardless of the CT findings. Furthermost, UGIC studies provided the extra benefits of delineation the shape of the sleeve, give an idea about the sites, sizes, and numbers of leak openings, and assist the surgeon in planning his next intervention (endoscopy or surgery) [2]. UGIC studies resulted in a detection rate close to CT in diagnosing the leak.

### Limitations

Up to the authors’ knowledge, this study shares the largest number of clinical presentations, laboratory, and radiological findings of patients with post LSG leak treated at a single center. Also, no previous papers studied the numbers of misdiagnoses nor ER visits for leak patients. The retrospective design is a limitation of this paper. Six patients leaked after band removal to revisional LSG. Unfortunately, data of either one or two steps band to sleeve were not available. Additionally, data of false positive radiological imaging and the details of the surgical techniques of patients who had been operated outside our center were not available.

## Conclusion

Abdominal pain, tachycardia, or fever after LSG should raise the suspicion of a leak. Most patients had abnormally high levels of WBCs and CRP but hypoalbuminemia in 76.2%. CT abdomen and UGIC study detected leaks in 75% and 77.5% consecutively. Only 29.3% of patients were diagnosed correctly as a leak from the first visit to the ER. The treating surgeons or ER physicians should rely on their clinical sense and their role is pivotal in early detection of the leak.
